# Using tools to engage health-care facilities (HCFS) in a global movement: World Health Organisation (WHO) Save Lives: Clean Your Hands (SL: CYHS) annual campaign

**DOI:** 10.1186/1753-6561-5-S6-P264

**Published:** 2011-06-29

**Authors:** C Kilpatrick, J Somner, B Allegranzi, E Mathai, S Bagheri Nejad, D Pittet

**Affiliations:** 1 Patient Safety, World Health Organisation , Geneva, Switzerland; 2 Ophthalmology, Norfolk and Norwich University Hospitals NHS Foundation Trust, Norwich, UK; 3 WHO Collaborating Centre, University of Geneva Hospitals, Geneva, Switzerland

## Introduction / objectives

Running a successful campaign is challenging. At a global level, SL:CYHs aims to engage all HCFs. The use of specific WHO tools was evaluated to understand if HCFs would also take action at the point of care around an annual campaign day.

## Methods

In 2010, two new tools were issued on the WHO website: the Moment 1 tool and Hand Hygiene Self-Assessment Framework (HHSAF). Electronic communications were primarily used to promote their use to a large database of contacts. Web site access and download information were gathered by analysing WHO web statistics with Urchin software (Google Inc.).

## Results

From April-June 2010 the Moment 1 tool was downloaded 7 693 times, a mean of 101/day.The HHSAF wasdownloaded 27 526 times from its launch on 5 May 2010 to end Feb 2011. The existing WHO 'how to handwash' poster was the only tool more popular at 45 978 downloads since launched in 2009. For a Moment 1 global observation survey, the first of its kind, more than 300 SL:CYHs registered HCFs from 47 countries took part. For the HHSAF, it is estimated that over 2 000 registered HCFs will submit data to WHO in 2011.

## Conclusion

Despite reflecting different time periods, other than the 'how to handwash' poster downloads, which were potentially increased by the H1N1 pandemic, the HHSAF is the most popular download. The Moment 1 global survey in 2010 improved WHO’s understanding of HCFs' move from commitment to action in support of this global campaign and local change; the HHSAF has facilitated further engagement of a considerable number of HCFs through. A fledgling global campaign can reach a wide audience and the availability of tools can support this.

## Disclosure of interest

None declared.

